# CULTIVATING CULTURAL COMPETENCE WITH INTERDISCIPLINARY TEAMS IN SERIOUS ILLNESS CARE

**DOI:** 10.1093/geroni/igad104.2345

**Published:** 2023-12-21

**Authors:** Karen Bullock, Kim Stansbury

**Affiliations:** Boston College, Boston, Massachusetts, United States; NC State University, Raleigh, North Carolina, United States

## Abstract

A workforce of gerontologists that is prepared to go beyond cultural humility to achieve baseline levels of awareness, skills, and transferrable knowledge to address racial disparities is necessary for creating equity in serious illness care with older adults. Cultural competence, the complex integration of awareness, knowledge, and skills enhances one’s capacity to engage effectively across cultural and racial groups to achieve equitable outcomes. Research Objectives The goal of this research was to design and assess outcomes of an innovative curriculum using simulation and role-play to train practitioners how to deliver patient-centered, goal concordant care with patients and families of racial groups that have been historically excluded from equitable healthcare in the U.S. Procedures: Interdisciplinary competency-based curriculum was developed and facilitated by an interdisciplinary team of educators and researchers. Advanced practice social workers were key members of serious illness care teams. With specialty training, they were prepared to be leaders in teaching learners how to conduct psychosocial assessments and interventions that beyond superficial levels of curiosity to applying competency-based practices with racially and culturally diverse populations. Implications: Addressing the impacts of the COVID19 pandemic in gerontology research and practice with racially diverse patients and families warrants a social justice framework for designing healthcare interventions that are inclusive of populations that have been most adversely impacted due inequities woven into the fabric of our society. Conclusion: This cultural competence training (CCT) program was effective in preparing learners to address racial inequities in healthcare for patients and families living with serious illness.

